# The Gut Microbiota of Healthy Chilean Subjects Reveals a High Abundance of the Phylum Verrucomicrobia

**DOI:** 10.3389/fmicb.2017.01221

**Published:** 2017-06-30

**Authors:** Sayaka Fujio-Vejar, Yessenia Vasquez, Pamela Morales, Fabien Magne, Patricia Vera-Wolf, Juan A. Ugalde, Paola Navarrete, Martin Gotteland

**Affiliations:** ^1^Laboratory of Microbiology and Probiotics, Institute of Nutrition and Food Technology, University of ChileSantiago, Chile; ^2^Department of Nutrition, Faculty of Medicine, University of ChileSantiago, Chile; ^3^Centro de Genética y Genómica, Facultad de Medicina, Clínica Alemana Universidad del DesarrolloSantiago, Chile; ^4^Microbiology and Mycology Program, Institute of Biomedical Sciences, Faculty of Medicine, University of ChileSantiago, Chile

**Keywords:** Chilean gut microbiota, healthy normal weight, 16S rRNA gene sequencing, bacterial communities, fecal samples

## Abstract

The gut microbiota is currently recognized as an important factor regulating the homeostasis of the gastrointestinal tract and influencing the energetic metabolism of the host as well as its immune and central nervous systems. Determining the gut microbiota composition of healthy subjects is therefore necessary to establish a baseline allowing the detection of microbiota alterations in pathologic conditions. Accordingly, the aim of this study was to characterize the gut microbiota of healthy Chilean subjects using 16S rRNA gene sequencing. Fecal samples were collected from 41 young, asymptomatic, normal weight volunteers (age: 25 ± 4 years; ♀:48.8%; BMI: 22.5 ± 1.6 kg/m^2^) with low levels of plasma (IL6 and hsCRP) and colonic (fecal calprotectin) inflammatory markers. The V3-V4 region of the 16S rRNA gene of bacterial DNA was amplified and sequenced using MiSeq Illumina system. 109,180 ± 13,148 sequences/sample were obtained, with an α-diversity of 3.86 ± 0.37. The dominant phyla were Firmicutes (43.6 ± 9.2%) and Bacteroidetes (41.6 ± 13.1%), followed by Verrucomicrobia (8.5 ± 10.4%), Proteobacteria (2.8 ± 4.8%), Actinobacteria (1.8 ± 3.9%) and Euryarchaeota (1.4 ± 2.7%). The core microbiota representing the genera present in all the subjects included *Bacteroides*, *Prevotella*, *Parabacteroides* (phylum Bacteroidetes), *Phascolarctobacterium*, *Faecalibacterium*, *Ruminococcus*, *Lachnospira*, *Oscillospira*, *Blautia*, *Dorea*, *Roseburia*, *Coprococcus*, *Clostridium*, *Streptococcus* (phylum Firmicutes), *Akkermansia* (phylum Verrucomicrobia), and *Collinsella* (phylum Actinobacteria). Butyrate-producing genera including *Faecalibacterium*, *Roseburia*, *Coprococcus*, and *Oscillospira* were detected. The family Methanobacteriaceae was reported in 83% of the subjects and *Desulfovibrio*, the most representative sulfate-reducing genus, in 76%. The microbiota of the Chilean individuals significantly differed from those of Papua New Guinea and the Matses ethnic group and was closer to that of the Argentinians and sub-populations from the United States. Interestingly, the microbiota of the Chilean subjects stands out for its richness in Verrucomicrobia; the mucus-degrading bacterium *Akkermansia muciniphila* is the only identified member of this phylum. This is an important finding considering that this microorganism has been recently proposed as a hallmark of healthy gut due to its anti-inflammatory and immunostimulant properties and its ability to improve gut barrier function, insulin sensitivity and endotoxinemia. These results constitute an important baseline that will facilitate the characterization of dysbiosis in the main diseases affecting the Chilean population.

## Introduction

The gastrointestinal tract is colonized by trillions of microorganisms known as the gut microbiota, which includes more than 1,000 different bacterial species. The two predominant phyla, Firmicutes and Bacteroidetes, represent about 90% of the microbial population (Qin et al., 2010). This microbial community has coevolved with humans for hundreds of thousands of years, establishing a symbiotic relationship with their host and performing essential activities considered as vital. It contributes to the extraction of energy and nutrients from foodstuffs, synthesis of vitamins, development of the immune system and protection against pathogens (Qin et al., 2010). Alterations of the microbial interactions with the host affect the gut barrier function as well as the local immune system, resulting in the disruption of the intestinal homeostasis and contributing to the development of several human diseases including gastrointestinal disorders (inflammatory bowel diseases (IBD), diarrheic syndrome, colorectal cancer, etc.), autoimmune diseases (multiple sclerosis, type-1 diabetes, rheumatoid arthritis), metabolic diseases (obesity, type 2 diabetes, non-alcoholic hepatic steatosis, atherosclerosis) and neurological disorders (autism, Parkinson’s disease) (Bravo et al., 2012; Julio-Pieper et al., 2014; Jandhyala et al., 2015). In fact, the gut microbiota composition of the individuals affected by these non-communicable diseases is altered and characterized by a decrease in microbial diversity (Konturek et al., 2015). For these reasons, the gut microbiota is currently considered as a new target to improve patient care through therapeutic (specific antibiotics, fecal transplant) or dietary intervention (newly designed probiotics or prebiotics) allowing the return to a “healthy” gut microbiota (González-Arancibia et al., 2016). Determining the composition of the gut microbiota of healthy subjects is therefore necessary to establish a baseline that can help us to understand the association between altered gut states and diseases.

Various factors are known to affect the taxonomic composition of the gut microbiota, making it difficult to define a shared core microbiota common to all individuals in a population. Microbiota can vary among individuals or populations according to host genetics, dietary habits, age, ethnic origin, geographic location and lifestyle (Zoetendal et al., 1998; Turnbaugh et al., 2008, 2009; De Filippo et al., 2010; Arumugam et al., 2011; Yatsunenko et al., 2012; Claesson et al., 2012; Cotillard et al., 2013; Schnorr et al., 2014; Suzuki and Worobey, 2014). For example, it has been hypothesized that variations in the gut microbiota may be observed in individuals or animals living in colder regions that need to extract more energy and to store more fat compared to those living in warmer regions (Suzuki and Worobey, 2014). Nevertheless, it is difficult to distinguish the effects of these multiple elements in the composition of the gut microbiota when different populations are compared. In addition, most of these studies did not consider that diet represents one of the most important factors affecting the gut microbiota. Collectively, it is more plausible that there is no core microbiome shared by all individuals, but rather multiple types that differ according to the geographic location and lifestyle of human populations.

Most of the studies on the human gut microbiota to date have focused on populations from North America and Europe. Rapid changes in lifestyle and socioeconomic conditions in association with an epidemiological transition have occurred in Latin-American countries in the last decades (Albala et al., 2001; Vio et al., 2008), which are probably accompanied by progressive changes in the composition of the gut microbiota of their inhabitants (Magne et al., 2016). New data on microbiome composition are becoming available from Latin American populations, but we are still orders of magnitude away from the knowledge available for other human groups from other continents (Escobar et al., 2014; Clemente et al., 2015; Carbonetto et al., 2016). In Chile, where the incidence of chronic metabolic diseases associated with gut dysbiosis is increasing, there is no available data about the microbiota composition of healthy or diseased subjects. For these reasons, this study describes the first characterization of the gut microbiota of healthy Chilean subjects using 16S rRNA gene sequencing.

## Materials and Methods

### Subject Enrollment Criteria

The study protocol was approved by the Ethics Committee for Research in Humans of the Institute of Nutrition and Food Technology (INTA), University of Chile, in compliance with the Helsinki Declaration. Asymptomatic, normal weight (BMI between 19.2 and 24.7 kg/m^2^) volunteers of both genders, aged 18–39 (**Table 1**), living in Santiago city and who had provided a signed, informed consent, were recruited in the study. Exclusion criteria included smoking, pregnancy and history of gastrointestinal, autoimmune, neurological and/or chronic metabolic diseases. Subjects with treatments interfering with intestinal permeability, motility or microbiota, such as dietary treatment for weight loss or drug intake (antibiotics, anti-inflammatory drugs, laxatives or prokinetics) during the month preceding the study were also excluded. Health status was confirmed through biochemical and lipid profiles and by the absence of colonic and systemic inflammation as suggested by fecal calprotectin and plasma Il-6 and high-sensitivity C-reactive protein (hsCRP), respectively (**Table 1**), as previously described (Morales et al., 2016). The subjects consumed their habitual diet and during the week before stool sampling, they were counseled by two registered dietitians to standardize their daily intake of macronutrients and avoid excess of dietary fats. The daily intake of energy (kcal) and macronutrients (% of caloric intake) of the subjects, as determined by a 48-h dietary recall, was as follows: (Means [CI_95%_]): energy 2078 [1946–2210]; carbohydrates: 58.9 [56.5–60.8]; proteins 15.2 [14.8–16.1] and lipids: 25.9 [23.9–27.9], according to the recommended dietary allowances. The subjects also consumed 19.9 g/day [18.3–21.6] of dietary fiber.

**Table 1:** Anthropometric, biochemical and clinical data of the 41 Chilean volunteers (Means ± SD).

Volunteers characteristics	
Female/male	20/21
Age (years)	25.0 ± 4.2
Height (m)	1.66 ± 0.10
Weight (kg)	62.2 ± 7.8
Body mass index (kg/m^2^)	22.5 ± 1.6
Glycemia (mg/dl)	89.8 ± 10.3
Total cholesterol (mg/dl)	166 ± 31
High density lipoproteins (mg/dl)	54.1 ± 12.9
Low density lipoproteins (mg/dl)	93.1 ± 28.3
Triglycerides (mg/dl)	101 ± 33
High-sensitivity C-reactive protein (hsCRP) (mg/l)	1.5 ± 2.4
Interleukin-6 (pg/ml)	8.0 ± 1.7
Fecal calprotectin (μg/g of stool)	12.3 ± 13.6


### Sampling, DNA Extraction and Identification of the Fecal Microbiota

Fecal samples were collected by the 41 volunteers in sterile plastic containers and stored at -30°C until further processing. Genomic DNA was extracted from 220 mg of stool samples using the QIAmp DNA Stool Mini Kit (Qiagen, Hilden, Germany) according to manufacturer instructions. Library preparation and Illumina sequencing were performed at the Roy J. Carver Biotechnology Center, University of Illinois at Urbana-Champaign, Champaign, IL, United States. In brief, library preparation was performed using the Fluidigm Access Array (Fluidigm, South San Francisco, CA, United States), in which 2 ng of DNA measured with a Qubit 2.0 fluorometer (Thermo Fisher Scientific, Carlsbad, CA, United States) were amplified in a two-step process. In the first step, the V3–V4 region of the 16S rRNA gene was amplified using the primers 341F (5′-CCTACGGGNGGCWGCAG-3′) and 785R (5′-GACTACHVGGGTATCTAATCC-3′) (Klindworth et al., 2013). The index and sequencing adaptors required were added in a second PCR. The amplicons obtained were quantified through Qubit fluorometry and their sizes verified for 11 random samples using an Agilent 2100 Bioanalyzer (Agilent Technologies, Santa Clara, CA, United States) to determine their overall quality. Amplicons were then pooled in equimolar concentration and purified with a 2% agarose e-gel (EX 2% agarose, Invitrogen, Life Technologies, Grand Island, NY, United States). The cleaned pool was reanalyzed with the Bioanalyzer to verify the effectivity of the clean-up step and to determine the average size of the amplicons. Finally, the pooled libraries were quantified with qPCR performed using a CFX connect Real-Time PCR (Bio-Rad, Hercules, CA, United States) to achieve an accurate quantification, before loading the libraries to the sequencer. Sequencing was performed with MiSeq Illumina system (Illumina, San Diego, CA, United States), using the V3 kit, generating 2×300 nt paired end reads.

### Sequence Processing, Comparison against Other Microbiome Datasets

De-multiplexing of the reads was performed using the software CASAVA v. 1.82. Paired reads were merged using FLASH (V1.2.11) with default parameters (Magoč and Salzberg, 2011), adaptors removed using Cutadapt (V1.9) (Martin, 2011), and quality trimmed using Trimmomatic (V0.36) (Bolger et al., 2014). To be able to compare this dataset with other studies that targeted different regions, we used a closed-reference strategy. First, 16S rRNA gene sequence data used in this study were obtained from: (1) Human Microbiome Project (HMP) data set (Human Microbiome Project Consortium, 2012)^1^, (2) Argentina (Carbonetto et al., 2016), (3) Colombia (Escobar et al., 2014), (4) Hadza population from Tanzania (Schnorr et al., 2014), (5) Italy (Schnorr et al., 2014), (6) South Korea (Nam et al., 2011), (7) Matses ethnic group, Peru (Obregon-Tito et al., 2015), and (8) Papua New Guinea (Martínez et al., 2015). These studies targeted different regions of 16S rRNA, but all the studies shared either the V3 and/or V4 regions, sequenced using either the 454 or Illumina platform. Paired-end reads were processed as previously mentioned (Masella et al., 2012). For all datasets, chimeric sequences were filtered using VSEARCH (Rognes et al., 2016) against the Greengenes database (DeSantis et al., 2006; 13_8 database). OTUs were built using the closed reference protocol in QIIME (V 1.8.0) (Caporaso et al., 2010) using the Greengenes 13.8 database, at 97% sequence similarity (Mcdonald et al., 2012). All abundance and diversity analyses were done using Phyloseq (McMurdie and Holmes, 2013). Rarefactions curves for the Chilean subjects were calculated using QIIME, subsampling in the range of 10–17,000 sequences, with a step of 1,000 sequences, and 10 re-samplings on each step. Values were obtained for the observed OTUs and Chao1 index.

To test for association of microbial abundance (at the genus level) with clinical metadata we performed a multivariate analysis using Multivariate Analysis by Linear Models (MaAsLin) version for R. MaAsLin^2^ performs boosted, additive general linear models between metadata and microbial abundance. Boosting of metadata and selection of a model was performed per taxon. All microbial data were arcsine-square root transformed. The metadata used were age, sex, body mass index (BMI), glycemia, triglycerides, total cholesterol, fecal calprotectin, and plasma interleukin-6 and hsCRP level. Associations were considered significant below a *q*-value threshold of 0.25 as recommended by the authors and as used in previous microbiome studies (Tong et al., 2014; Lim et al., 2016).

Data analysis was done using R (R Core Team, 2015). Calculation of alpha and beta diversities was done using Phyloseq (McMurdie and Holmes, 2013). Sample alpha diversity was calculated using the Shannon index. Differences in alpha diversity between genders for the Chilean subjects were determined using Kruskal–Wallis test. Comparisons between different countries were visualized using principal coordinate analysis (PCoA) of the weighted UniFrac distances between samples (Lozupone et al., 2006). Ellipses were drawn into the visualization using a 95% confidence interval. Evaluation of differences between countries, based on the beta diversity values, was done using ADONIS. A test for homogeneity of multivariate dispersions was applied to evaluate if these differences were due to group dispersions. Both analyses were done using the Vegan package in R. Linear discriminant analysis (LDA) effect, implemented in LEfSe (Segata et al., 2011), was used to compare the relative abundance of the different taxa between all the groups. An alpha value of 0.05 was used for the ANOVA and Wilcoxon tests to identify features with significantly different abundances between assigned taxa compared to other populations. A size-effect threshold of 4.0 on the logarithmic LDA score was used for discriminative microbial taxa. A one-against-all test was used to compare each different country of origin, against the rest of the dataset.

## Results

**FIGURE 1:**
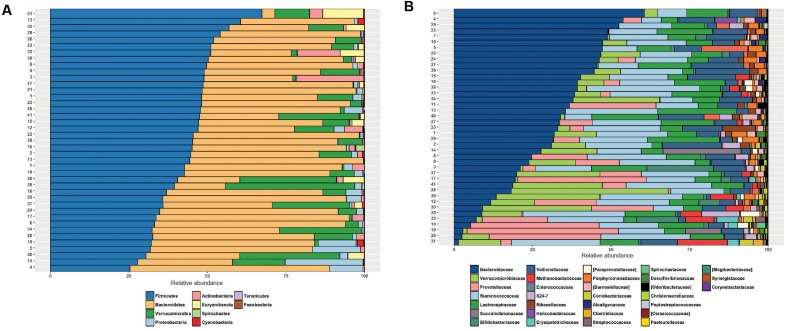
Fecal microbiota composition of healthy, normal weight Chilean subjects by sequencing the V3–V4 of 16S rRNA gene using the MiSeq Illumina system. Relative abundance (%) of phyla **(A)** and families **(B)** identified in healthy normal weight Chileans (*n* = 41).

The anthropometrical, biochemical and clinical characteristics of the volunteers are described in **Table 1** and Supplementary Table S1 (Supplementary Data). The fecal microbiota of the volunteers was characterized through sequencing the V3–V4 region of the 16S RNA gene. A total of 4,476,399 high-quality filtered sequences were obtained, i.e., 109,180 ± 13,148 sequences per sample. The rarefaction curves reached an asymptote, which indicated that the sequence depth was sufficient to represent the majority of bacterial community diversity (Supplementary Figure S1). Overall, 13 phyla and 57 families were detected (**Figures 1A,B**) and an α-diversity (Shannon index) of 3.86 ± 0.37 was estimated. No differences in α-diversity were observed between genders (*p* = 0.1362, Supplementary Figure S2). The most abundant phyla were Firmicutes (43.6 ± 9.2%) and Bacteroidetes (41.6 ± 13.1%); followed by Verrucomicrobia (8.5 ± 10.4%), Proteobacteria (2.8 ± 4.8%), Actinobacteria (1.8 ± 3.9%) and Euryarchaeota (1.4 ± 2.7%). Euryarchaeota corresponds to the only identified phylum belonging to the Archaea domain. The other 7 phyla identified (Cyanobacteria, Tenericutes, Fusobacteria, Lentisphaerae, Spirochaetes, Synergistetes, and TM7) had relative abundance <0.2%. The phyla Firmicutes, Bacteroidetes, Verrucomicrobia, Proteobacteria, and Actinobacteria were detected in all subjects. High inter-individual variability was detected in the microbiota composition: the relative abundances ranged from 25.3 to 67.3% for Firmicutes, 4.1–63.5% for Bacteroidetes, 0.002–41.2% for Verrucomicrobia and 0.05–24.9% for Proteobacteria (**Figure 1A**).

Bacterial families were dominated by Bacteroidaceae (30.2 ± 16.1%), Ruminococcaceae (19.4 ± 6.7%), Lachnospiraceae (10.4 ± 5.6), Verrucomicrobiaceae (9.4 ± 11.7), Prevotellaceae (8.8 ± 13.1%) and Veillonellaceae (7.8 ± 4.5%) (**Figure 1B**). These families were detected in all subjects. Porphyromonadaceae, Methanobacteriaceae, Rikenellaceae, Bifidobacteriaceae and S24-7 showed relative abundances ranging from 1 to 2%. The other bacterial families had relative abundances <1%. Only small differences were detected between women and men in the abundance of bacterial families, affecting two families with abundances <0.5%: Streptococcaceae (♀: 0.27 ± 0.65% vs. ♂: 0.096 ± 0.22%; *p* = 0.015) and Pasteurellaceae (♀: 0.060 ± 0.080% vs. ♂: 0.018 ± 0.035%; *p* = 0.007).

The genera identified in all subjects, representing the core microbiota, includes *Bacteroides*, *Prevotella, Parabacteroides* (Bacteroidetes phylum), *Phascolarctobacterium*, *Faecalibacterium*, *Ruminococcus*, *Lachnospira*, *Oscillospira*, *Blautia*, *Dorea*, *Roseburia*, *Coprococcus*, *Clostridium*, *Streptococcus* (Firmicutes phylum), *Akkermansia* (Verrucomicrobia phylum), and *Collinsella* (Actinobacteria phylum). We observed a dominance of *Bacteroides* over *Prevotella* in 34/41 (83%) of the subjects. The *Methanobacteriaceae* family, belonging to the phylum Euryarchaeota of the Archaea domain was detected in 83% (34/41) of the subjects, and *Desulfovibrio*, the most representative sulfate-reducing genus, in 76% (31/41) of the subjects. Some genera including pathogens were also detected: *Treponema* in 5 individuals, *Campylobacteraceae* (with reads assigned to the genera *Arcobacter* and *Campylobacter*) in 15 subjects, and *Helicobacter* in 2 subjects. To identify specific microbial taxa significantly associated with clinical metadata, we performed a multivariate association with linear models (MaAsLin) analysis. Of all the variables, only glycemia showed a significant negative association with genus *Haemophilus* (Pasteurellaceae family) (*p*-value: 2.18E^-4^, *q*-value = 0.153, Supplementary Figure S3).

**FIGURE 2:**
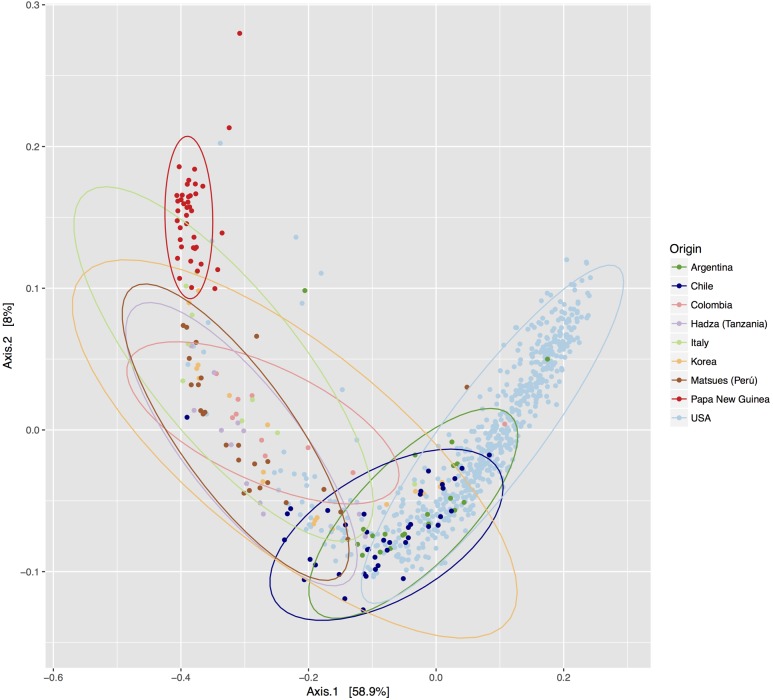
Comparison of the Chilean gut microbiota versus gut microbiota of individuals from other geographic locations. PCoA of the beta diversity values based on weighted Unifrac distances. Each color represents a population from a specific geographic area. Ellipses were drawn using a confidence interval of 95% for each group.

**FIGURE 3:**
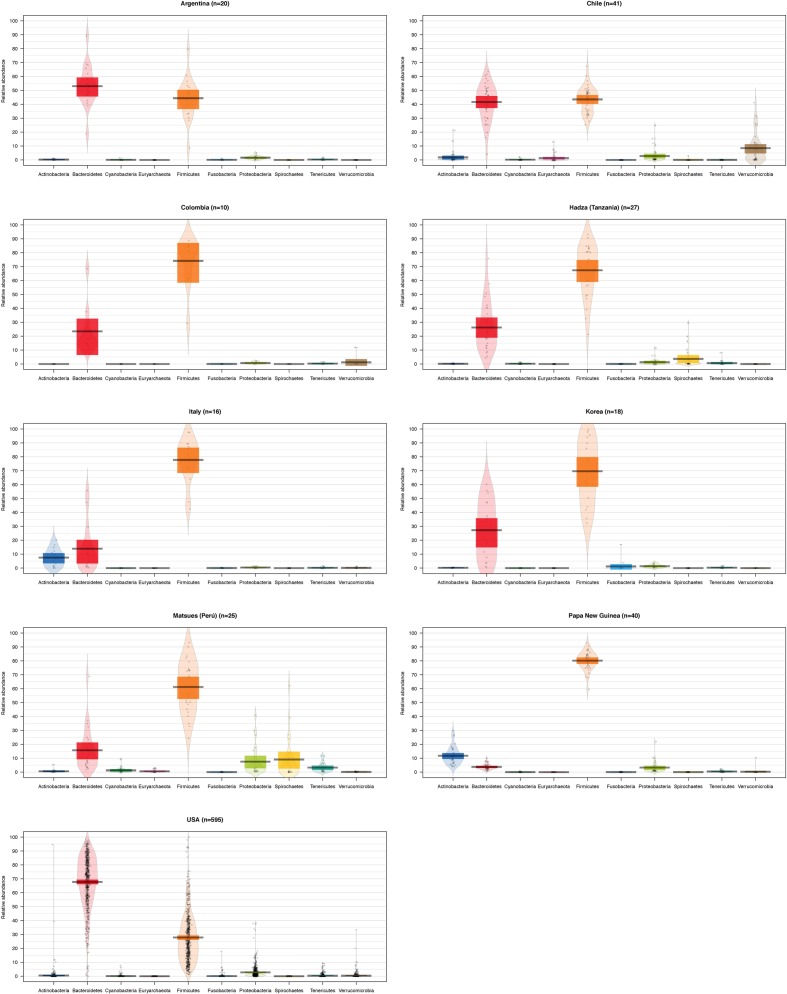
Abundance of the main bacterial phyla identified in the gut microbiota of individuals from different geographic locations. Violin plots showing the abundance of the most abundant bacterial phyla for the gut microbiota of different populations. The number of subjects for each population is shown on the title, and each bar represents a different bacterial phylum. The black line indicates the median of the values, the colored box the interquartile range and the area in light color corresponds to the distribution of the data.

**FIGURE 4:**
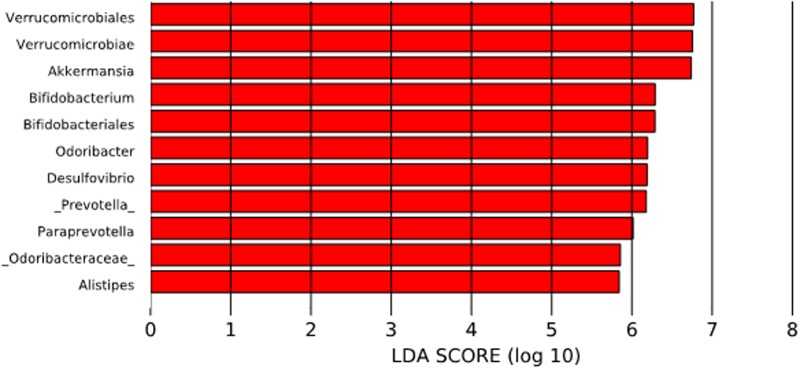
Linear discriminant analysis (LDA) Effect Size (LEfSe) analysis of bacterial taxa present in the Chilean subjects and those from other countries. The significantly enriched populations are shown only for the Chilean population.

To compare the differences between microbial compositions among the different populations, we analyzed the beta diversity of populations, using weighted UniFrac distances (**Figure 2**). The microbiota of the United States and that of the Papua New Guinea subjects were clearly segregated while those from the other populations were distributed between them. The microbiota of the Chilean subjects significantly differed from that of Papua New Guinea (*p* = 0.001) and the Matses ethnic group (*p* = 0.001) and did not differ from the subjects of the other countries, being closer to the populations of Argentina and some sub-populations from the United States. We also evaluated the contribution of component PC3 showing only 4.3%. A visualization of the same dataset using a Bray–Curtis distance metric, showed a similar distribution of the different populations (Supplementary Figure S4). The difference between populations seems to be explained by differences in the abundance of phyla, as described in **Figure 3**, where the most dominant were Bacteroidetes and Firmicutes. More particularly, the Papua New Guinea microbiota showed a high dominance of Firmicutes over Bacteroidetes while the contrary was observed in the United States population. With a higher Firmicutes/Bacteroidetes ratio, the gut microbiota of Colombia, Italy, South Korea, Hadza and Matses ethnic groups were more similar to the Papua New Guinea microbiota. The Chilean and Argentinean gut microbiota harbored the same relative abundance of Firmicutes and Bacteroidetes phyla. However, the composition of the gut microbiota from Chilean subjects had greater abundance of members of the phylum Verrucomicrobia. We then evaluated significant differences in relative abundance of bacterial orders, families and genera of the Chilean subjects compared to the other populations through LDA Effect Size (LEfSe). We observed that the Chilean population was more enriched in Verrucomicrobiales, Verrucomicrobiae and *Akkermansia*, Bifidobacteriales and *Bifidobacterium*, Odoribacteraceae and *Odoribacter*, *Paraprevotella*, *Prevotella*, *Alistipes*, and *Desulfovibrio* (**Figure 4**).

Regarding the relative abundance of potentially pathogenic bacterial genera, no differences were observed between countries for *Helicobacter*. The abundance of *Treponema* in the Matses and Hadza populations was significantly higher (*p* < 0.002 and *p* < 0.006, respectively) than that reported for all the other countries, including Chile (Supplementary Figure S5A). For *Campylobacter*, no significant differences were observed between populations (Supplementary Figure S5B).

## Discussion

This is the first report describing the taxonomic composition of the gut microbiota from young, healthy, normal weight, Chilean subjects. Although the great majority of the studies describing the composition of gut microbiota in different human populations are carried out from fecal samples, it is important to note that the human fecal microbiota is an unreliable reflection of the cecal or colonic microbiota. Marteau et al. (2001) showed, for example, that facultative anaerobes represent only 1% of total bacteria in the feces versus 25% in the cecum and that, more particularly, lower levels of *Bifidobacterium*, *Bacteroides* and members of the *C. coccoides* and *C. leptum* groups were detected in the cecum than in stool samples. This is obviously an important limitation that must be considered in the interpretation of the results.

Our results showed high inter-individual variability, despite the highly homogenous characteristics of the subjects (in terms of age, weight, biochemical and lipid profiles, absence of inflammation). Similar observations were also reported in other normal weight populations (Nam et al., 2011; Human Microbiome Project Consortium, 2012; Escobar et al., 2014; Schnorr et al., 2014; Obregon-Tito et al., 2015; Carbonetto et al., 2016). In most of our subjects we observed a dominance of *Bacteroides* over *Prevotella*. This enterotype was first shown by Wu et al. (2011) to correspond to individuals consuming western diets rich in animal proteins and fat, in contrast to fiber-rich diets that favor the presence of *Prevotella*. During the last 30 years Chile has experienced a rapid epidemiological and nutritional transition characterized by the gradual adoption of an energy-rich western-type diet accompanied by a shift from infectious to chronic diseases (Atalah et al., 2014). For example, the intake of calories from fat increased from 21 to 28% between 1980 and 1998; the daily intake of meat and meat-derived products is currently about 150 g (Albala et al., 2002; Ministerio de Salud, 2011). The modern urban Chilean diet has also been incorporating increasing amounts of processed foods, refined sugar and salt while decreasing the intake of fruits and vegetables. As a result, the prevalence of obesity in adults and children and type 2 diabetes is among the most elevated in the world. The volunteers of our study consumed this type of diet, rich in animal fat and proteins, but with a moderate daily caloric intake (∼2078 Kcal/day), explaining their normal weight. These findings could explain the dominance of *Bacteroides* over *Prevotella* in their microbiota. An appropriate characterization of the nutrient sources in studies of microbiota composition could improve the identification of specific nutrient-taxa associations.

In addition to *Bacteroides*, the bacterial core also includes taxa normally identified in the human gut microbiota such as the succinate-utilizing *Phascolarctobacterium* (Watanabe et al., 2012) and some beneficial butyrate-producing genera such as *Faecalibacterium*, *Roseburia*, *Coprococcus*, and *Oscillospira* (which have never been cultivated and are related to lean status and health) (Konikoff and Gophna, 2016). *Collinsella*, which belongs to the phylum Actinobacteria, has been found to be significantly increased in type 2 diabetes compared to prediabetic and non-diabetic American subjects (Lambeth et al., 2015). The presence and role of this microorganism in the Chilean gut microbiota should be explored in more detail in larger studies in the future. Interestingly, we detected a high proportion (>8%) of the mucus-degrading genus *Akkermansia*, belonging to the phylum Verrucomicrobia. This genus has been suggested to be a potential biomarker of a healthy gut status. It has been proposed that the growth of this bacterium is favored by low availability of enteral nutrients such as in long-term fasting and malnutrition (Belzer and de Vos, 2012).

Regarding the Archaea domain, several groups were detected in the microbiota of our subjects. The *Methanobacteriaceae* family of the phylum Euryarchaeota was observed in 83% (34/41) of the subjects. All of them harbored members of the genus *Methanobrevibacter*, in agreement with the high prevalence of this group in the adult population (>50%). *Methanobrevibacter smithii*, the most dominant methanogen in the human gut, is implicated in the production of methane through the reduction of CO_2_ using H_2_ (or formate) (Gaci et al., 2014). We identified sequences that matched the genus *Methanosphaera* in one individual. One of the most important representatives of this group is *Methanosphaera stadtmaniae* that produces methane from methanol (Gaci et al., 2014). Methanogens of the order *Methanomassiliicoccales*, which participate in the reduction of methyl-compounds [mono, di and trimethylamine (TMA)] to produce methane (Gaci et al., 2014) were not identified in our study. This could be important, considering that TMA, produced through the metabolism of choline and L-carnitine by gut microorganisms, is subsequently oxidized in the liver into the pro-atherogenic trimethylamine oxide (TMAO) (Gaci et al., 2014). Based on this consideration, it has been hypothesized that dietary supplementation with these “Archaebiotics” could prevent cardiovascular diseases in at-risk subjects (Gaci et al., 2014).

*Desulfovibrio*, the most representative sulfate-reducing genus, was detected in 76% (31/41) of the subjects. Although a high prevalence (>50%) of this bacterium has been reported in patients with IBD, it can exert pro- or anti-inflammatory effects depending on the concentration of hydrogen sulfide (H_2_S) produced in the gut lumen (Linden, 2014).

Another interesting taxon identified in our study was *Treponema*, which was detected in five individuals (12.5%). The genus *Treponema* belongs to the phylum *Spirochaetes* and includes human pathogenic and non-pathogenic species. Non-pathogenic *Treponema* has been described as part of the normal microbiota of the intestine, oral cavity and genital flora and was shown, for example, to be exclusively present in rural African children compared to European children (De Filippo et al., 2010). Recent studies using high-throughput sequencing of the 16S rRNA gene showed a high prevalence of *Treponema* in the gut of human populations consuming polysaccharide-rich diet, probably due to the fact that some *Treponema* species hydrolyze cellulose and xylans. Our results show that the relative abundance of this genus in the Chilean population was clearly lower than that described in the Matses and Hadza, hunter-gatherer populations consuming high amount of dietary fiber, especially from tubers (Schnorr et al., 2014).

Furthermore, we detected 15 (37.5%) subjects colonized with enteric pathogens belonging to the family *Campylobacteraceae* (*Arcobacter* and *Campylobacter)*, and 2 subjects with *Helicobacter. Campylobacter jejuni* is the leading global cause of foodborne disease associated with the consumption of contaminated chicken in the developed world, and with contaminated water in the developing countries (Kaakoush et al., 2015; Johnson et al., 2017). Asymptomatic fecal carriage of the pathogen has been reported in humans (Dicksved et al., 2014; Kampmann et al., 2016). The high level of asymptomatic *Campylobacter* carriage observed in our study could be related to the high incidence (38–68%) of this microorganism in Chilean poultry meat (Figueroa et al., 2009), which is the main source of animal protein consumed in Chile. Other evidence reinforcing this hypothesis is the fact that some isolates from human campylobacteriosis and broiler meat are genetically indistinguishable by pulsed-field gel electrophoresis (PFGE) (González-Hein et al., 2013). *Helicobacter pylori*, a bacteria residing in the stomach, has been linked to several gastric diseases such as stomach cancer. It is estimated that more than 50% of the population is colonized with this microorganism worldwide, and over 80% of these infected humans remain asymptomatic (Peleteiro et al., 2014). *H. pylori* is highly prevalent in the Chilean population (Gotteland et al., 2006). Although the classical methods to detect this bacterium are culture of gastric biopsy or a urea breath test, *H. pylori* can also be detected in stool samples (Talarico et al., 2016).

We also explored possible correlation between the microbiota and metadata of the Chilean subjects. We only detected a significant association between the basal glycemia and *Haemophilus*: at higher relative abundance of *Haemophilus*, lower basal glycemia is observed. This data are in line with observations reported in other studies showing higher relative abundances of *Haemophilus* in healthy subjects compared to patients with glucose intolerance (Zhang et al., 2013) and with a type 2 diabetes mellitus group (Qin et al., 2012).

Finally, we compared our data to those obtained from public datasets generated from other populations. The results suggest that the diversity of the Chilean gut microbiota is similar to that of the Argentina and United States subpopulations, while it clearly differs from those from Papua New Guinea and the Matses ethnic groups. Further studies with greater coverage and power are needed to confirm these differences. It is interesting to note that, although the selection of our subjects was controlled as closely as possible in terms of age, health status, lifestyle and dietary intake, the dispersion of the abundances values was quite high. This could reflect differences in genetic background, particularly the Amerindian heritage whose importance may vary from one subject to another in the Chilean population. The composition of the microbiota of the Chileans and Argentinians appears to be closer because their lifestyle and diet are similar and the proportion of Amerindian heritage is lower than in other Latin American countries such as Colombia or Peru, where diet is also different. In the case of the United States subjects, it must be stated that the number of samples is clearly higher than those from the other countries, as well as their dispersion. This probably also reflects the heterogeneity of these subjects in terms of genetic background and the existence of other factors contributing to the microbiota composition.

An important difference in the Chilean subjects compared to populations from other countries was the higher abundance of phylum Verrucomicrobia. This phylum is a member of the PVC (Planctomycetes-Verrucomicrobia-Chlamydiae) superphylum which includes phylogenetically related bacteria with unusual characteristics such as the existence of a complex and dynamic endomembrane system that, in some aspects, makes them closer to eukaryotic cells. It includes a small number of genera isolated from fresh water, soil and animal feces; *Akkermansia muciniphila* is the main member of this phylum identified in humans (though the presence of *Luteolibacter* or *Verrucomicrobium* was also reported in three subjects from United States and Matses ethnic groups). *A. muciniphila* is a strict anaerobe, Gram-negative, mucus-inhabiting bacterium which is considered as a true symbiont due to its wide distribution in the animal kingdom. In humans, it is detected in the intestine of most healthy individuals where it represents 1–4% of the total microbiota (Collado et al., 2007); it is one of the bacteria on which the enterotype classification is based (Arumugam et al., 2011). It is a highly specialized bacterium capable of using mucins as a sole source of carbon and nitrogen (Derrien et al., 2004) and stimulating mucin expression and mucus secretion through a positive feedback (Derrien et al., 2010). This specialization would represent a competitive advantage in case of nutrient deprivation such as fasting, malnutrition or total parenteral nutrition. As a result of mucin degradation, *A. muciniphila* releases acetate and propionate which are easily available for the host (Derrien et al., 2004, 2011). *A. muciniphila* colonizes the human gut early in life (1 month) and increases rapidly with age, reaching 10^8^/g of stool in adults (Collado et al., 2007). Its abundance is lower in patients with IBDs (Png et al., 2010) or appendicitis (Swidsinski et al., 2011), as well as in animal models of obesity and obese humans (Zhang et al., 2009; Santacruz et al., 2010). This is an interesting finding, considering that this agent also exhibits anti-inflammatory and immunostimulant properties. Increasing the intestinal counts of *A. muciniphila* in these subjects through the administration of polyphenols or prebiotics results in the improvement of barrier function, endotoxemia and insulin sensitivity (Anhê et al., 2015), the increased expression of intestinal Fiaf (a circulating inhibitor of lipoprotein lipase) and RegIII (a bactericidal C-type lectin targeting Gram-positive bacteria) (Anhê et al., 2015), and higher intestinal content of bioactive lipids participating in the endocannabinoid system. Interestingly, the abundance of *A. muciniphila* is higher after antibiotic administration (Dubourg et al., 2013) and in patients treated with metformin; this could represent one of the mechanisms of action by which this drug improves glucose homeostasis and insulin resistance in diabetic patients (Everard et al., 2013; Shin et al., 2014).

It is unclear why the Chilean subjects displayed higher abundance of Verrucomicrobia considering that none of them were consuming antibiotic or metformin (exclusion criterion) and their intake of dietary fiber (with eventual prebiotic properties) was not especially elevated. Accordingly, the presence of this taxon needs to be studied further to confirm the current results, to determine the factors subjacent to this high abundance and to evaluate its role in the gut microbiota of healthy subjects.

## Conclusion

The Chilean gut microbiota shows high inter-individual variability despite the highly homogenous characteristics of the subjects. The differences between the Chilean gut microbiota and those of other populations seem to be associated with differences in the Firmicutes/Bacteroidetes ratio and the high abundance of Verrucomicrobia. These results constitute an important baseline to explore further the possible alterations of gut microbiota composition occurring in the most prevalent diseases in the Chilean population, and the host factors implicated in inter-individual variability.

## Data Access

Raw datasets are available in the European Nucleotide Archive (ENA) under the accession number PRJEB16755.

## Author Contributions

PM, PN, and MG designed the study. PM performed the recruitment of the volunteers, collection of stool samples and the extraction of fecal bacterial DNA. SF-V and JU processed the raw sequences. SF-V, YV, PV-W, and JU performed the bioinformatic and statistical analysis. SF-V, YV, PV-W, JU, FM, PN, and MG analyzed the results and wrote the manuscript.

## Conflict of Interest Statement

The authors declare that the research was conducted in the absence of any commercial or financial relationships that could be construed as a potential conflict of interest.

## Footnotes

1 http://hmpdacc.org/HM16STR/healthy/

2 https://huttenhower.sph.harvard.edu/maaslin
