# Supplementary material for: The Gut Microbiota of Healthy Chilean Subjects Reveals a High Abundance of the Phylum Verrucomicrobia
Source: Front Microbiol. 2017 Jun 30;8:1221. doi: 10.3389/fmicb.2017.01221 (PMC5491548; doi:10.3389/fmicb.2017.01221)
Supplement: Supplementary file 1 [file Table_1.PDF]

Table S1: Metadata of the Chilean subjects

Volunteer	Age (years)	Sex	Height (m)	Weight (Kg)	BMI (kg/m^2)	Glycemia (mg/dl)	Total cholesterol (mg/dl)	HDL (mg/dl)	LDL (mg/dl)	Tryglicerides (mg/dl)	hsCRP (mg/l)	Il-6 (pg/ml)	Fecal Calprotectin (µg/g of stool)
**1**	33	Female	1,62	60,5	23,05	101,5	189,0	57,1	111,2	103,4	2,0	8,5	3,87
**2**	28	Female	1,64	59,6	22,16	94,8	188,5	56,9	112,2	97,3	2,2	9,0	6,19
**6**	22	Female	1,59	50,1	19,82	90,8	163,0	66,3	77,9	94,3	3,2	9,2	4,59
**7**	28	Female	1,71	59,8	20,45	88,8	158,5	46,3	90,4	108,7	1,8	7,8	23,43
**10**	20	Female	1,54	45,8	19,31	95,2	138,0	50,8	75,0	60,8	0,6	8,8	5,12
**12**	27	Female	1,74	67,5	22,29	94,5	150,5	50,4	68,2	159,6	2,3	8,7	3,20
**13**	22	Female	1,61	57,2	22,07	94,7	170,2	64,2	76,5	147,5	0,2	9,0	4,48
**14**	21	Female	1,57	58,2	23,61	83,2	122,0	57,4	47,3	86,7	1,5	7,8	6,78
**15**	39	Female	1,63	65,2	24,54	94,0	164,3	32,0	111,7	103,4	1,3	9,3	10,58
**16**	28	Female	1,60	53,4	20,99	85,6	184,3	85,0	83,6	78,3	2,6	6,7	17,57
**18**	23	Female	1,57	47,3	19,19	94,4	185,2	71,7	83,9	148,2	1,9	7,0	9,92
**19**	19	Female	1,57	56,8	23,04	90,3	146,3	54,1	79,4	64,6	0,4	7,5	4,10
**20**	18	Female	1,52	55,4	24,14	91,7	118,1	60,6	42,7	74,5	2,1	8,7	15,00
**23**	19	Female	1,63	55,5	20,89	91,7	143,4	71,0	45,6	134,6	0,4	6,3	10,70
**24**	27	Female	1,64	53,4	19,83	90,0	241,2	96,4	112,0	163,5	1,5	9,7	2,40
**25**	26	Female	1,57	56,8	23,04	84,4	154,5	60,2	72,8	107,3	3,8	10,3	39,57
**26**	25	Female	1,60	58,5	22,85	70,7	217,6	62,7	135,4	97,6	0,3	5,1	14,70
**28**	28	Female	1,55	55,2	22,98	80,1	185,7	45,2	118,9	108,1	1,3	6,2	4,27
**29**	27	Female	1,68	60,3	21,49	71,7	173,9	46,7	110,8	82,1	0,1	5,1	9,52
**32**	25	Female	1,57	48,7	19,76	70,7	174,5	63,5	86,1	124,4	3,7	8,0	3,90
**3**	24	Male	1,75	62,5	20,41	101,9	139,1	41,4	83,1	73,0	0,4	8,5	55,20
**4**	28	Male	1,76	73,5	23,73	116,5	145,5	45,2	82,2	90,5	0,4	8,5	55,71
**5**	24	Male	1,74	68,9	22,76	94,2	168,0	50,6	99,5	89,7	0,7	8,5	14,64
**8**	27	Male	1,72	67,8	22,92	100,5	132,0	57,3	61,0	68,4	13,1	11,2	7,70
**9**	26	Male	1,66	58,7	21,30	110,2	134,0	39,9	113,0	104,9	2,1	9,8	29,53
**11**	21	Male	1,71	70,5	24,11	82,5	126,0	55,3	60,8	49,4	0,0	9,5	7,60
**17**	23	Male	1,66	68,2	24,75	71,1	155,1	66,0	76,8	61,6	0,1	6,7	4,06
**21**	23	Male	1,68	67,1	23,75	89,3	196,4	48,1	128,6	98,8	0,4	6,3	1,45
**22**	20	Male	1,71	71,3	24,53	97,7	170,2	42,9	98,9	142,2	0,5	6,1	5,62
**27**	24	Male	1,61	62,1	23,96	83,2	152,7	45,6	89,7	87,0	0,0	5,8	10,02
**30**	28	Male	1,71	67,0	22,91	85,7	152,7	44,1	95,0	68,3	0,4	5,1	20,90
**31**	31	Male	1,83	80,8	24,26	108,9	177,9	51,9	101,1	124,8	0,3	6,6	3,46
**33**	24	Male	1,77	67,7	21,61	77,9	220,5	34,3	162,5	118,7	0,2	8,7	3,50
**34**	31	Male	1,71	61,3	20,96	81,6	129,7	34,9	79,2	78,0	0,5	6,6	2,07
**35**	18	Male	1,81	68,1	20,79	94,7	155,1	45,2	92,5	87,0	7,7	10,3	39,57
**36**	24	Male	1,71	71,3	24,38	76,9	142,7	55,7	76,0	55,3	0,5	6,1	6,10
**37**	26	Male	1,70	69,2	23,94	88,8	265,3	55,3	184,7	126,5	0,6	12,7	2,58
**38**	24	Male	1,73	71,8	24,13	93,9	176,7	49,9	105,6	105,6	0,3	8,9	4,52
**39**	23	Male	1,65	61,8	22,84	91,0	179,8	52,3	109,2	91,6	0,2	7,8	19,80
**40**	26	Male	1,67	68,2	24,45	89,2	179,8	39,5	99,3	205,0	0,1	8,3	5,26
**41**	25	Male	1,72	67,0	22,65	89,2	154,0	64,4	77,8	59,3	0,2	7,2	4,49
**Average**	**25**		**1,66**	**62,2**	**22,5**	**89,8**	**166,4**	**54,1**	**93,1**	**100,7**	**1,5**	**8,0**	**12,3**
**min**	**18**		**1,52**	**45,8**	**19,2**	**70,7**	**118,1**	**32,0**	**42,7**	**49,4**	**0,0**	**5,1**	**1,4**
**max**	**39**		**1,83**	**80,8**	**24,7**	**116,5**	**265,3**	**96,4**	**184,7**	**205,0**	**13,1**	**12,7**	**55,7**
**SD**	**4,2**		**0,1**	**7,8**	**1,6**	**10,3**	**31,2**	**12,9**	**28,3**	**33,4**	**2,4**	**1,7**	**13,6**
