# Supplementary material for: The Gut Microbiota of Healthy Chilean Subjects Reveals a High Abundance of the Phylum Verrucomicrobia
Source: Front Microbiol. 2017 Jun 30;8:1221. doi: 10.3389/fmicb.2017.01221 (PMC5491548; doi:10.3389/fmicb.2017.01221)
Supplement: Supplementary file 2 [file Image_1.PDF]

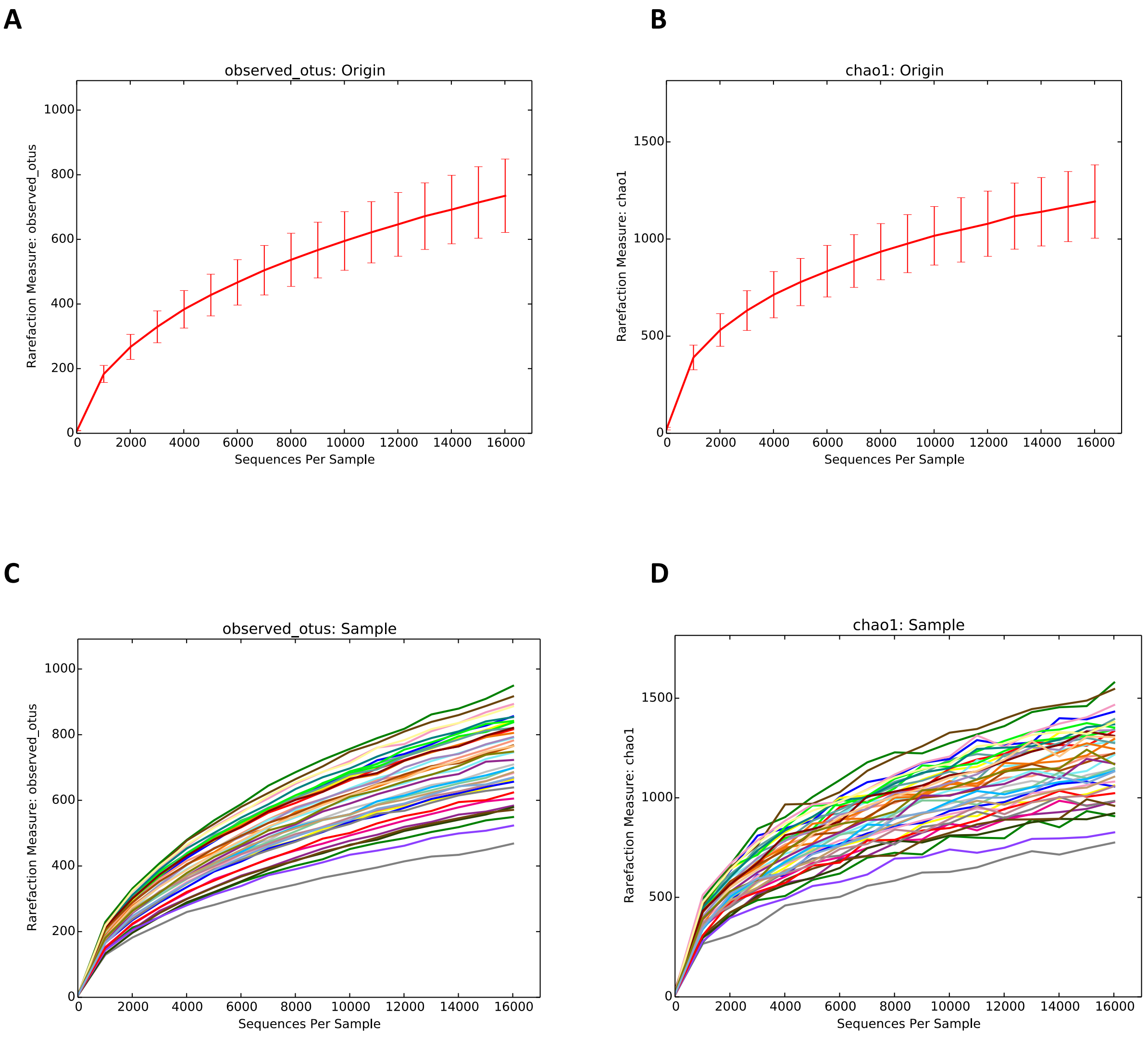

Figure S1. Rarefaction curves corresponding to the samples from the Chilean subjects. **A** and **B,** shows the summary of all the Chilean samples for the observed OTUs and using the chao1 index. **C** and **D** shows the individual samples corresponding to each subject, both for the observed OTUs and chao1. Values were determined using QIIME, using a range of 10-17,000 sequences, with a step of 1,000, and 10 calculations for each step.
