# Supplementary material for: The Gut Microbiota of Healthy Chilean Subjects Reveals a High Abundance of the Phylum Verrucomicrobia
Source: Front Microbiol. 2017 Jun 30;8:1221. doi: 10.3389/fmicb.2017.01221 (PMC5491548; doi:10.3389/fmicb.2017.01221)
Supplement: Supplementary file 3 [file Image_2.PDF]

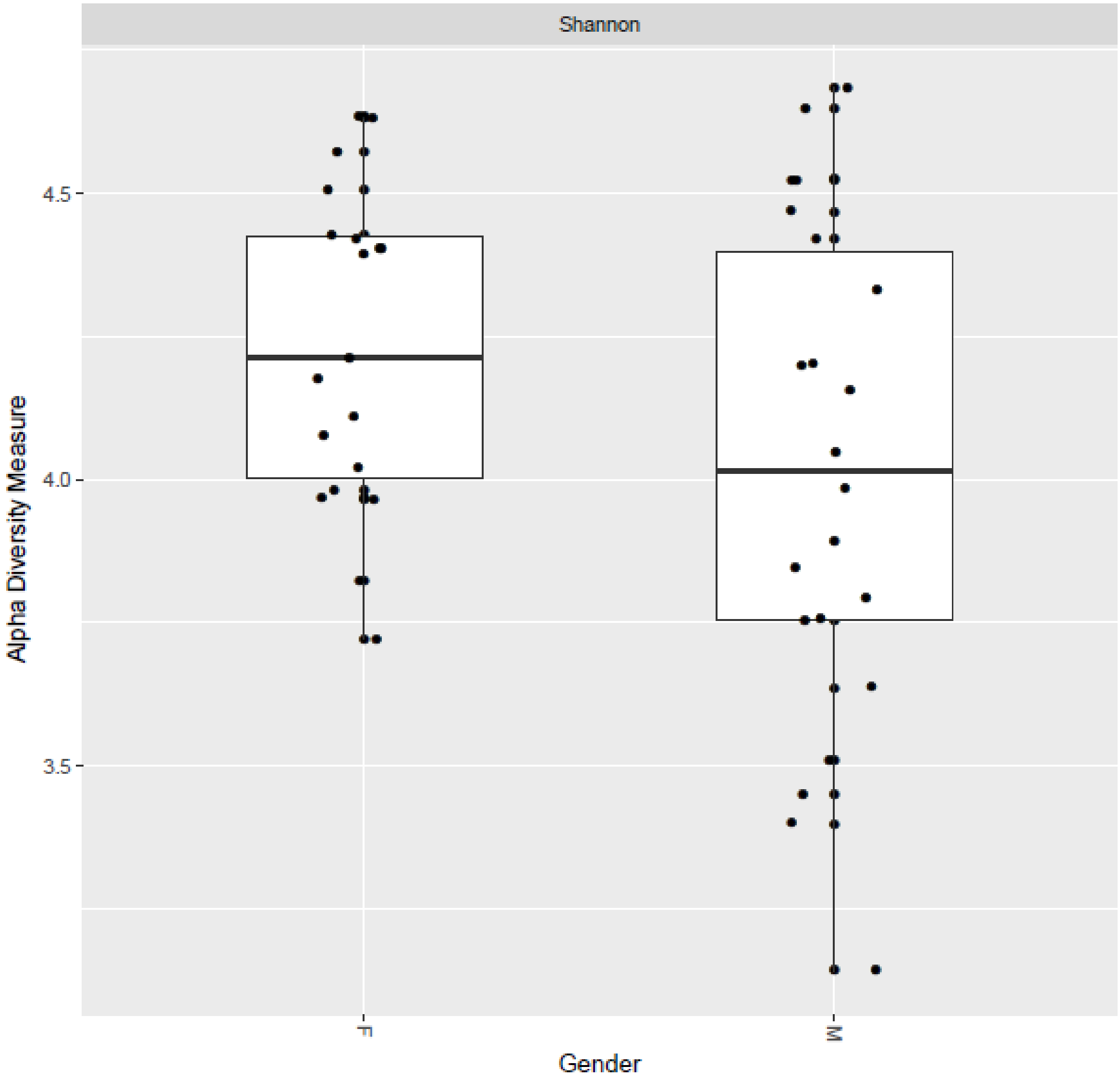

Figure S2: alpha-diversity of the fecal microbiota of Chilean subjects, by gender. No significant difference was observed (p=0.14).
