# Supplementary material for: The Gut Microbiota of Healthy Chilean Subjects Reveals a High Abundance of the Phylum Verrucomicrobia
Source: Front Microbiol. 2017 Jun 30;8:1221. doi: 10.3389/fmicb.2017.01221 (PMC5491548; doi:10.3389/fmicb.2017.01221)
Supplement: Supplementary file 4 [file Image_3.PDF]

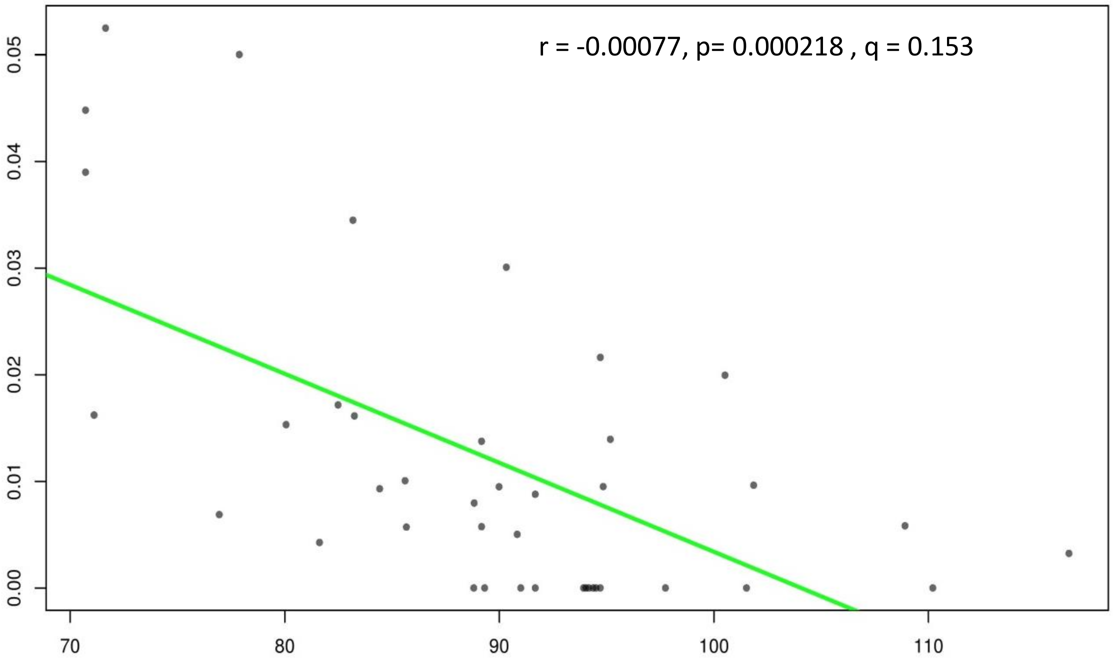

Figure S3: MaAsLin scattergram showing significant correlation between glycemia levels and the relative abundance of *Haemophilus*. The corresponding correlation coefficient, p and q value are shown in the Figure.
