# Supplementary material for: The Gut Microbiota of Healthy Chilean Subjects Reveals a High Abundance of the Phylum Verrucomicrobia
Source: Front Microbiol. 2017 Jun 30;8:1221. doi: 10.3389/fmicb.2017.01221 (PMC5491548; doi:10.3389/fmicb.2017.01221)
Supplement: Supplementary file 5 [file Image_4.PDF]

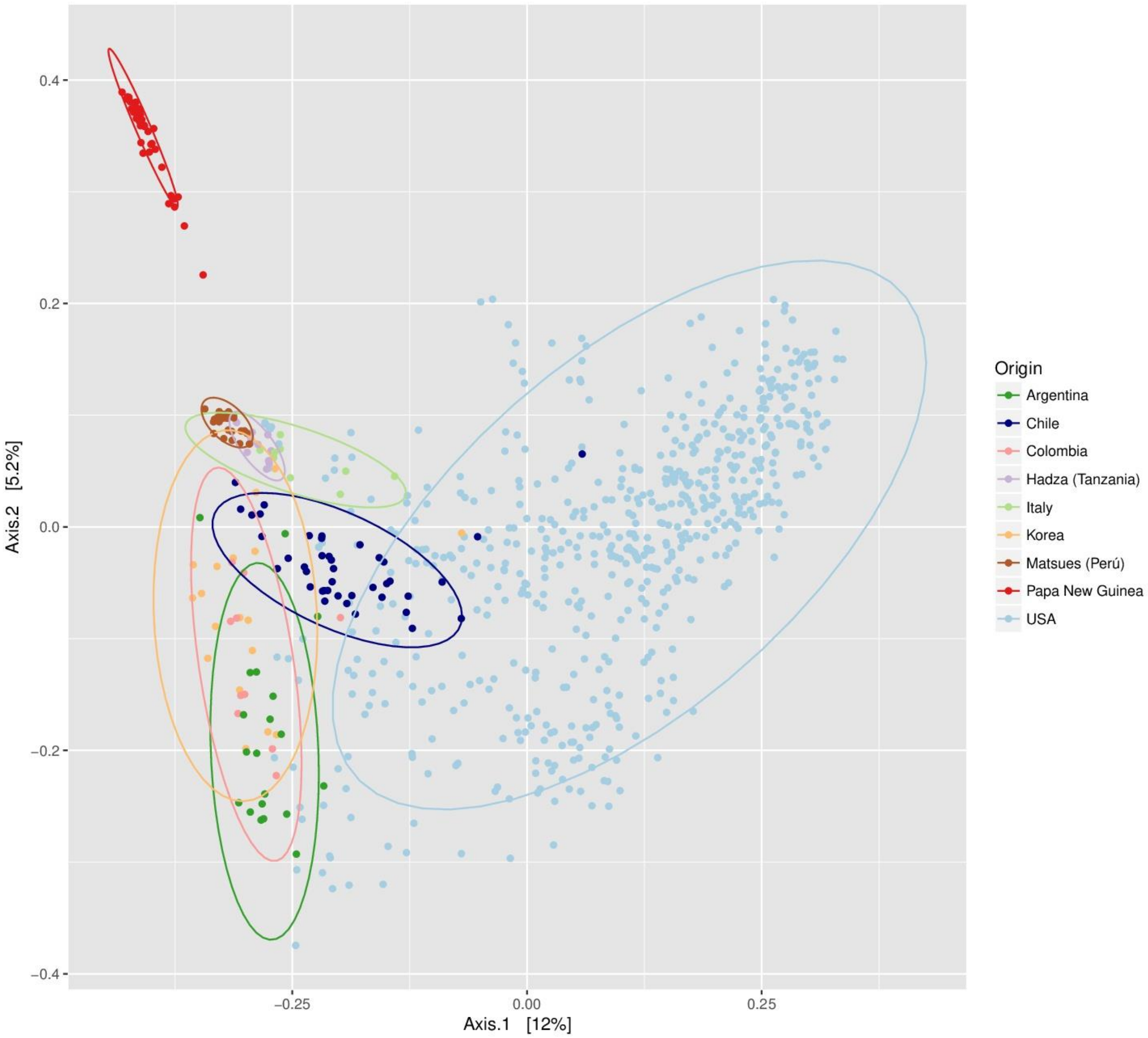

Figure S4: Comparison of the Chilean gut microbiota versus gut microbiota of individuals from other geographic locations. PCoA of the beta diversity values based on Bray-Curtis distance metric. Each color represents a population from a specific geographic location. Ellipses were drawn using a confidence interval of 95% for each group.
