# Supplementary material for: The Gut Microbiota of Healthy Chilean Subjects Reveals a High Abundance of the Phylum Verrucomicrobia
Source: Front Microbiol. 2017 Jun 30;8:1221. doi: 10.3389/fmicb.2017.01221 (PMC5491548; doi:10.3389/fmicb.2017.01221)
Supplement: Supplementary file 6 [file Image_5.PDF]

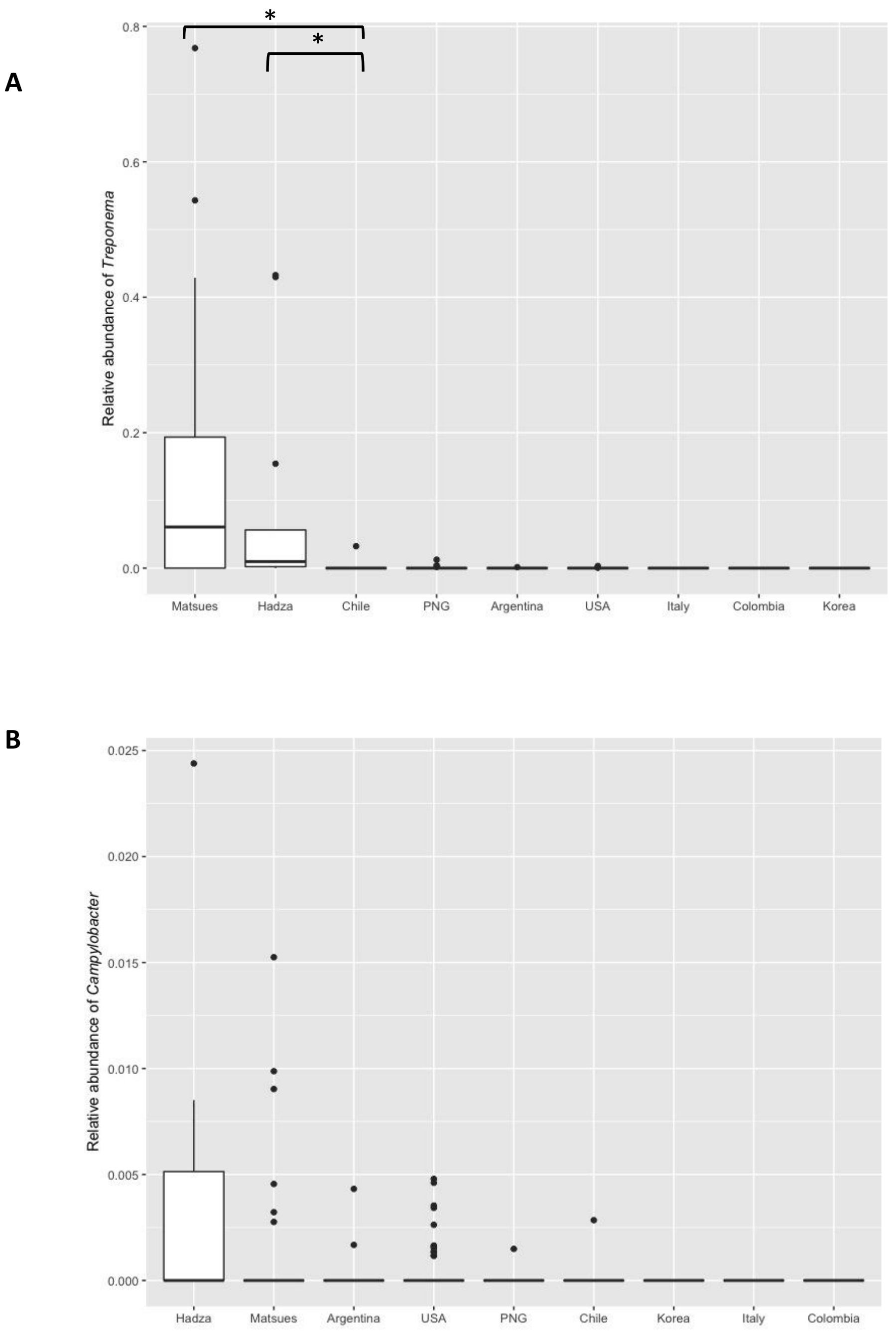

Figure S5 : Relative abundance of *Treponema* (A) and *Campylobacter* (B) in the different populations. Comparisons between countries were performed by Kruskall Wallis test (*p<0,004 and q<0,025 (FDR adjusted p-value)). The significant differences are shown only for the Chilean population.
